# An Enquiry into the Efficacy of Digitalis in the Treatment of Idiopathic Epilepsy

**Published:** 1841-04

**Authors:** 


ART. V.—*An Enquiry into the Efficacy of Digitalis in the treatment of Idiopathic Epilepsy.* By EDMOND SHARKEY, A.B. M.D. One of the Lecturers on Midwifery in the Hunterian School of Medicine, Charlotte-street Bloomsbury.—*London*, 1841. 8vo, pp. 80.

THE contents of this volume might perhaps have formed as appropriate a subject for communication to one of the journals as for a separate treatise. In order to make a book, the author has been obliged to introduce a general description of Epilepsy, something after the manner common in inaugural dissertations. This we think superfluous. We must add that many of his remarks on the pathological relations of epilepsy, and the operation of remedies, are so vague and inconsecutive, that it is extremely difficult to perceive their drift. He endeavours to establish some analogies, especially one between sleep and epilepsy, in which we cannot say we think he is very successful, and with which we deem it unnecessary to detain the reader. Leaving, therefore, the theoretical part of Dr. Sharpey's treatise, as not demanding any particular notice, we may state the following as the chief practical points on which he insists with reference to the exhibition of digitalis in epilepsy.

1. That it is only to the idiopathic and uncomplicated forms of the disease, that the remedy is generally applicable.

2. That in the treatment of such forms of epilepsy, digitalis has had as much success, in proportion to the number of trials made, as nitrate of silver, or oil of turpentine, and has succeeded in cases in which these have failed.

3. That the best mode of exhibiting it is according to the following formula, used by the author's father.

℞. Foliorum Digital. purp recentium. ℥iijss.

Contunde in mortario in pulpam; dein adde cerevisiæ fortioris, lb.j. Infunde per horas septem; dein cola exprimendo. Capiat liquoris colati ℥iv, cum pulv. fol. Polypodii quercus siccatorum, aut radicis siccatæ gr. x.

4. That its efficacy probably depends on a specific power, and not merely on its effect upon the circulation.

5. That what is called the *cumulative* effect of digitalis amounts simply to this: that a certain quantity must be taken before any effect is produced; that this is no other than the aggregate effect of the separate doses that have been taken; and hence, that there is much less danger than might be supposed in administering a very large dose of digitalis at once.

6. That the system may assume a state of *tolerance* of digitalis, as of tartar emetic, and other medicines.

7. That the treatment of epilepsy with digitalis should be commenced immediately after a fit, not immediately before one.

According to Dr. Sharkey, the earliest notice of the efficacy of digitalis in epilepsy is to be found in Parkinson's Theatrum Botanicum, London, 1640. The next writer who mentions this use of it, is Withering. After him, Dr. Currie employed the medicine in three cases; and in the year 1807, it was tried by our author's father in conjunction with Dr. Mills of Dublin. Dr. Sharkey, senior, published his cases in the Lancet of May 27, 1831; and his son, in the treatise before us, has given an abstract of them, with additional cases derived from other sources. The cases are not very clearly detailed, and we are left much in the dark as to the intervals at which the remedy was administered. We dismiss the subject with a recommendation to our brethren to make a fair trial of the powers of digitalis in epilepsy.

